# Demonstrating consensus in argumentative settings: Co-constructions in children’s peer discussions

**DOI:** 10.1007/s10212-024-00840-7

**Published:** 2024-05-17

**Authors:** Judith Kreuz, Martin Luginbühl

**Affiliations:** 1 Centre of Oral Communication, University of Teacher Education Zug, Zug, Switzerland; 2 German Studies, University of Basel, Basel, Switzerland

**Keywords:** Oral argumentation skills, Co-constructions, Synchronization, Peer group interaction

## Abstract

‘Taking part’ in conversations requires different activities from the interactants depending on the kind of conversation. This article investigates co-constructions in oral peer group discussions of elementary school children from grades 2 to 6 (7–12 years old). Although dissent is the starting point of argumentations, negotiating processes in oral argumentations are often co-constructed by two or more speakers on different levels, including consensual contexts. Co-constructions presuppose that the second speakers recognize structures and expectations based on the turn of the first speaker and that they are able to complete or expand these structures. Therefore, co-constructions can be understood as an indicator for oral skills and as a key site of ‘taking part’ in small group discussions. The article will discuss two different kinds of co-constructions (morpho-syntactical and argumentative-structural) based on 60 transcripts from a bigger corpus of 180 peer discussions. The analysis will show that these co-constructions can be understood as synchronizations of thinking and acting and to what extent they are an indicator of oral skills and play an important role in cooperative learning settings. The results are relevant in school contexts when it comes to assess oral argumentation of students. For teachers, they are helpful to elicit requirements for children’s argumentation skills and to design tasks conducive to learn to argue and develop assessment tools accordingly.

## Research context: Oral argumentation and co-constructions

### Oral argumentation as interactive processes

Oral argumentation skills are a core competence in our societies (Iordanou & Rapanta, 2021; Quasthoff et al., 2019) and have gained attention in many educational standards (Hauser & Luginbühl, 2017, p. 89; Rapanta et al., 2013, p. 484). While there are several empirical studies on preschoolers (Arendt, 2019b; Baines & Howe, 2010; Bose & Hannken-Illjes, 2018; Komor, 2010; Zadunaisky Ehrlich & Blum-Kulka, 2014) as well as middle and high school students (Andrews, 2009; Grundler, 2011; Heller, 2012; Kuhn et al., 2013; Morek, 2020; Quasthoff & Kluger, 2020), there are not many studies on elementary school children aged 7–12 (but see Anderson et al., 1997; Brandt & Höck, 2012; Goetz & Shatz, 1999; Huth, 2014; Stivers & Sidnell, 2016). This article takes a closer look at oral peer discussions amongst elementary school children within the approaches of conversation analysis and argumentation studies, focusing on the co-construction of arguments.

Although the starting point of (oral) argumentation is (at least a potential) dissent, all interactions and therefore conversations are an interactional achievement, a joint production of the interactants (Birkner et al., 2020; Ferrara, 1992; Levinson, 2013). While written argumentations consist of monologues or – e.g., in an online chat – single contributions that are only published when the single contribution is finished and one after the other (but not simultaneously) and that are written without the possibility to observe the writing process itself, oral face-to-face argumentations are organized differently. All interactants are (and must be) present and can observe the multimodal production of turns of the other participants. Argumentation studies often focus on argument schemes while fundamental characteristics of conversations like the sequentiality, temporality and interactivity of conversations (Imo & Lanwer, 2019; Schegloff, 2007) are neglected (Gülich & Mondada, 2008, p. 16–19; see for further overview Sidnell & Stivers, 2013). From a Conversation Analysis perspective (cf. Sect. “Data and method”), all activities are coordinated and synchronized by the interactants all the time and the interactants’ single turns must be fitted continuously to the local context, including movements of gaze, head, body, as well as gestures and intonation etc. (Bose & Hannken-Illjes, 2020; Jacquin, 2015). Thus, single turns have to be analyzed in their sequential context and against the backdrop of the way the interactants treat and interpret them themselves and as the result of a joint activity resulting in “order at all points” (Sacks, 1984, p. 22). Therefore, the (argumentative) status of a turn (e.g., if a turn is a proposition, an agreement, or a disagreement) relies on the local, sequential context in the conversation, the rhetorical means, linguistic forms, and multimodal resources used (we will not focus on multimodal resources but address them in the analyses). Like this, process-related and rhetorical conversational aspects shape the argumentation, and the argumentative structures are at the same time shaped by them; the product and the process of oral argumentation are interdependent (see for more details Luginbühl & Müller-Feldmeth, 2022) and the analysis must take into account all of the mentioned aspects in detail.

Based on current work on (children’s) oral argumentation (Arendt, 2019b; Bose & Hannken-Illjes, 2018; Grundler, 2011; Heller, 2012), we understand oral argumentation in conversations as a (predominantly, but not exclusively) verbal activity, as an interactive, emergent and situated process in which open questions, facts and positions with different validity claims are marked as negotiable and in which justifications are used to make validity claims or positions plausible (cf. Hauser & Luginbühl, 2017). Consequently, regarding argumentative competence, oral argumentation skills not only entail aspects of argumentation logic, argument schemes, or single argumentative moves, but also the interactive processing of argumentation in the mediality of oral face-to-face conversation and relationship work (Grundler, 2011; Morek, 2017; Mundwiler et al., 2017).

What can be observed in conversations is not what an individual is *able to do* (in the sense of a cognitively, individuum based, cross-situational potential), but what it *is doing* in very specific circumstances. It is therefore the performances (instead of potential capabilities) that can be reconstructed, or what Deppermann (2004) calls “factual competence” (p. 20, our translation), i.e., observable, actual behavior. These performances might be the ‘normal’ and tried way people do things, it must not be an ‘ideal’ way, but a way that leads to a solution of the communicative task without bigger problems. This ‘normal’ way can be reconstructed by looking for communicative patterns, it can also be partly reconstructed from directly and indirectly named norms of the participants (see Hauser & Luginbühl, 2015).

This has to be taken into account when analyzing oral argumentations skills in school contexts: Our methodological approach with the means of Conversation Analysis makes it possible to describe oral argumentation processes among children in detail on the basis of systematically observed conversations in learning contexts and thus to gain further perspectives on argumentation, beyond aspects of written argumentation that often serves as reference point (Mundwiler et al., 2017). Analyzing oral argumentation without a normative perspective may reveal specific forms of oral argumentation (e.g., “argumentative co-constructions”, see Sect. “Video analysis – co-constructions in small group discussions”), which may also be of interest for the school context. Oral argumentation competences have long been part of school curricula – but they are also a cross-curricular key competence and argumentation supports subject learning, see e.g., Baker et al. (2019) and Mercer (2009) – and should accordingly be promoted and transparently assessed by teachers. The prerequisite for this is to describe processes and products of oral argumentation through precise observations (= diagnostic competence of teachers) and to assess them for the current speaking situation in situ. An essential criterion here is appropriateness – and not a specific norm (see Hauser & Luginbühl, 2015). Conversation-analytical research on argumentation can thus contribute to better understand teaching and acquiring argumentative competences and make this research fruitful for the school context.

### Synchronization and ‘taking part’ in face-to-face interaction: ‘co-constructions’

Conversations can be described as a “joint activity” (Linell, 1998, p. 86), “joint action” (Clark, 1996, p. 18) or “joint production” (Ferrara, 1992, p. 207), as two or more people carry out a coordinated activity with each other. This presupposes the assumption of a synchronization in face-to-face interaction in the sense of Luckmann (1983): “In the reciprocal mirroring of a face-to-face encounter, two streams of consciousness, and two body-bound, inner times are synchronized into the intersubjective time of direct, social interaction” (p. 74).

This kind of synchronization becomes obvious if the joint activity is interwoven to such a degree that the verbal actions become a “unified flow of linguistic structures” (Günthner, 2012, p. 8, our translation), which is the case in collaboratively produced co-constructions, e.g., if one child finishes a sentence started by another child. We understand co-constructions of this kind as one type of synchronization. ‘Co-construction’ as a term is used for a broad array of processes that are jointly achieved, often leaving the meaning of the term “quite elliptical” (Jacoby & Ochs, 1995, p. 171). While the term has been of some importance in social sciences, literary studies, or anthropology, it was first used in linguistics for the joint production of syntactical structures in the context of jointly produced turns, e.g., in Günthner (2000, 2012), Lerner (1991), Ono and Thompson (1995), or Szczepek (2000a, 2000b). While this understanding of co-constructions is still dominant, as Drescher (2015) argues, a broader understanding of co-constructions is discussed in an edited volume by Dausendschön-Gay et al. (2015). The term is related to bigger interactional sequences on a macro level but remains in its definition quite vague as it says “the joint action of interaction partners to continue an interaction toward a goal” (p. 22, our translation) and is used as an umbrella term for joint conversational activities. While this allows to use the term in many different contexts, we prefer a more specific understanding for our analysis (see for a discussion Kreuz, 2021, p. 89-99). Nevertheless this also includes the consideration of macrostructural aspects of conversations, like the co-constructions in “communicative projects” (Linell, 1998, p. 218) or “discourse units” such as narrations (Ohlhus, 2014), explanations (Morek, 2012), or argumentations (Heller, 2012), going beyond single syntactical constructions, but include certain communicative jobs with specific forms and content (like constituting dissent, establishing an obligation to provide justifications, providing and challenging justifications etc. in the case of argumentations, see Heller, 2012; Quasthoff et al., 2017, p. 90). This concept of co-construction can be related to ‘taking part’ in children’s peer discussions, whereby we understand by ‘taking part’ the individual contributions children make in a joint achievement of a conversation. With a focus on oral argumentation skills, we believe that co-constructions are a case in point of a common achievement and therefore for an analysis and description of taking part in conversations.

We argue that it is helpful to relate the more content and action related, ‘argumentative-structural’ aspect of co-constructions to the more form-related, syntactical aspect. In fact, we argue with Günthner (2012) that these aspects cannot be fully separated, as also more content and knowledge-oriented aspects must be materialized in language, gestures, body movements or other signs. Co-constructions can range from “close syntactic fit […] to mere pragmatic acceptability only loosely based on properties of syntactic form” (Rieser, 2001, p. 4). This allows for a more concise understanding of co-constructions and combines aspects of macro level (discourse units) with aspects of micro level (syntactical structures ‘in execution’), as Family et al. (2015) or Gülich & Krafft (2015) argue.

In the following, we distinguish two kinds of co-constructions that make synchronizations visible. The first is on the morpho-syntactical level, the second on the argumentative-structural level. Co-constructions on the morpho-syntactical level can be found when two speakers produce one syntactic structure or a word together (Lerner, 2004), or if one speaker is repeating or reformulating a part or the end of the turn of another speaker (Auer & Pfänder, 2011). On the one hand, structures can be completed (i.e., the first part would remain elliptical without the co-constructed element), on the other hand co-constructions can be expanding the turn of another person by adding a syntactical complement like a subclause or another “clausal glue-on” (Couper-Kuhlen & Ono, 2007, p. 531f.). In every case, co-constructions do not stand alone but are the second of a first part.

**Fig. 1:**
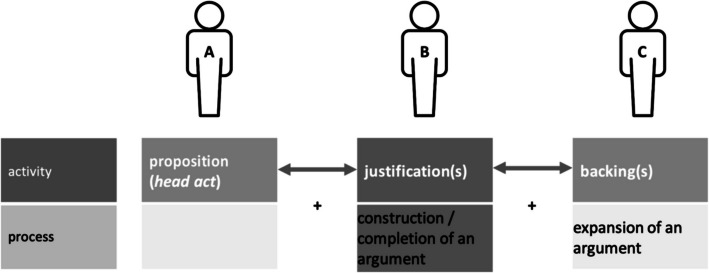
Possible structure of a co-construction on the argumentative-structural level (Kreuz, 2021, p. 143)

Co-constructions on the argumentative-structural level are completions of argumentative “head acts” (Kyratzis et al., 2010) like conclusions, statements, requests, proposals etc. with further argumentative elements to generate a complete argument. Argumentations consist of different structural elements, like the ones described by Toulmin (Toulmin, 2003 [1958], see also Kienpointner, 2008). They include claims (e.g., a proposition), grounds (e.g., evidence, facts), warrants (link between claims and grounds, e.g., a justification), backings (e.g., an example). Not all of them must be made explicit, often they are only implied, but can be reconstructed based on the local context. We speak of a co-construction on the argumentative-structural level when speakers produce an argumentative structure together across two (or more) different speakers’ turns (cf. also Kyratzis et al., 2010, p. 117). Figure 1 shows a possible structure of a co-construction in this sense.

A basic structure of a co-construction on the argumentative-structural level consists of a first part by speaker A, a proposition (e.g., a claim or an opinion). The second part from speaker B consists of the corresponding justification, completing the argument. Further, a third speaker can even elaborate the argument, e.g., by giving an example. In this case, we speak of an extended structure of a co-construction.

Co-constructions, be they morpho-syntactical or argumentative-structural, show that the second speaker recognizes a certain structure in the first part and that s/he can complete or expand this structure according to the expectations built up (Günthner, 2012). Speakers show like this not only a high involvement, but also specific oral skills, which are a prerequisite to recognize and complete/expand these structures. Co-constructions occur especially in *cooperative* (learning) settings and are always indicators for the oral skills in play. For both kinds of co-constructions also prosody and body movements, gaze etc. are important (Mondada, 2014; Szczepek Reed, 2011) and usually play an “accompanying” or “reinforcing” role in both forms of co-constructed argumentation, because oral communication and especially co-constructions cannot do without the multimodal orientation of the participants towards each other. As mentioned, this level of multimodality will not be in our focus but will be outlined in the analyses (especially Sect. “Expansion of argument structures “Lighter” – elaboration of argumentation”).

## Research questions

Within the context discussed so far, we are interested in the role the different co-constructions play in taking part in children’s peer discussions. We are especially interested in what role they play in solving communicative ‘problems’ or challenges, and what changes in these activities can be observed over time. Therefore, in addition to describing forms, it is necessary to ask about the pragmatic function of utterances or phenomena, in our case the function of a turn within an argumentative structure (e.g., justification, backing etc.). This leads us to the following research questions:

- Which synchronisation performances (especially on argumentative-structural level, but also on the morpho-syntactical level in form of shared syntax or completions of utterances) can be reconstructed in co-constructed negotiation processes within argumentative peer conversations and how do these co-constructions serve in taking part in the current conversation sequence?
- Which pragmatic functions of co-constructions can be reconstructed in the data and how is the ‘problem’ thereby processed/resolved?
- What differences can be observed between different ages?

In the last section (Sect. “Discussion”), we will also discuss which educational and didactic consequences can be derived from our results for the school context.

## Data and method

The following examples (Sect. “Video analysis – co-constructions in small group discussions”) are from a larger research project on oral argumentation skills of Swiss German elementary school children.^1^ In order to be able to reconstruct the ‘factual competence’ of the children, we collected a rather large corpus of 180 peer group discussions with 4 children each, 60 discussions each of grades 2, 4 and 6 of Primary School (7–12 years) (cf. Luginbühl et al., 2021) (cf. Table 1). The size of this corpus with 720 children allowed us to minimize the influence of factors like general language skills, sociodemographic and socioeconomic family status etc., that are hard to control.

**Table 1:** Corpus overview (Luginbühl et al., 2021, p. 187)

Grade	Topic/Setting	Consequence of Action	Abbreviation
Grade 2	20 × Robinson	no	Ro_K2
	20 × Donation	yes	Sm_K2
	20 × Donation	no	So_K2
Grade 4	20 × Robinson	no	Ro_K4
	20 × Donation	yes	Sm_K4
	20 × Donation	no	So_K4
Grade 6	20 × Robinson	no	Ro_K6
	20 × Donation	yes	Sm_K6
	20 × Donation	no	So_K6

The corpus also allows to reconstruct the systematic patterns of interaction, that is at the core of Conversation Analysis (see Sect. “Oral argumentation as interactive processes”). To get data as natural as possible, but that is rich and comparable at the same time (cf. Quasthoff, 2021), we developed three tasks similar to existing schoolbook tasks that made oral argumentation expectable (discussion about donation of fictive vs. real existing money and ‘Robinson-Crusoe’):

We did not give specific preparations (e.g., argumentation exercises, lists with possible arguments) and let the children discuss amongst themselves, with no adults present in the room when the instruction was done. We gave the three tasks we developed to 20 groups each from grades 2, 4, and 6. The examples discussed in this article are all taken from discussions of the same task, the ‘Robinson task’, as they have proven to be the more vivid and extensive ones. This made the data more suitable for our purposes of analysis. In this discussion task the children were asked to imagine that they were stranded on a desert island and had to select three objects out of a list of twelve to ensure their survival on the island. The task was to reach an agreement as a group, not just to convince someone. This has consequences for processing the argumentations, as jointly produced co-constructions are more common in our corpus than in dissentic contexts (see for the analysis of the entire Robinson corpus with roughly 5 times more co-constructions in consensual contexts than dissentic ones Kreuz, 2021; cf. Felton et al., 2015 for discussions’ framing). In the analysis, we therefore focus on the more common cases in our corpus, in which co-constructions occur in consensual contexts, in which dissent is at most latent. In our understanding based in linguistic conversation analysis and argumentation theory, we speak of a (at least temporal) consent, when propositions and/or justifications are explicitly (e.g. by agreeing, repeating the proposition/justification, giving additional justifications etc.) or implicitly (by not marking disagreement in any kind) agreed upon. We transcribed all videorecorded conversations using EXMARaLDA (https://exmaralda.org/de/) and the GAT 2 conventions (Selting et al., 2009).^2^

## Video analysis – co-constructions in small group discussions

Comparing grades 2, 4, and 6, we can see that the share of justifications per argumentative episode is augmenting, i.e., in a complex sequence of turns related to the argumentative treatment of a topic more justifications are given over time. While second graders produced justifications in 23% of all episodes, it was 35% in the fourth grade and 42% in the sixth (n = 2867, see Kreuz & Luginbühl, 2020). This comes along with an increasing complexity of these episodes. While in grade 2, argumentatively isolated statements prevail, longer, jointly produced episodes become more frequent in grades 4 and 6 (Luginbühl et al., 2021). While in the 2nd grade, the search for allies is often more important than giving justifications, in the 4th and 6th grade, the children claim for argumentative groundings in more and more cases. The children offer thereby also room for learning and practicing to each other (e.g., cf. Arendt, 2019a). It becomes also more frequent, that the children give justifications, when a consensus is already established (Kreuz, 2021).^3^ Mainly in these cases arguments are not only made by one person alone, but “across different speakers’ turns” (Kyratzis et al., 2010, p. 117). These so-called ‘co-constructions’, we will argue in the following, are a case in point when it comes to oral argumentations skills.

In this chapter, three examples of peer-group discussions are presented in which several levels of synchronization can be observed: According to our understanding, synchronizations of thinking and acting can be accessed by co-constructions (see Sect. “Research context: Oral argumentation and co-constructions”). In addition to co-constructions at the morpho-syntactical level (e.g., shared syntax and completions of utterances), the focus will be on co-constructions at the argumentative-structural level, where argumentative elements are completed and extended (see Sect. “Research context: Oral argumentation and co-constructions”). The selected examples are exemplary cases of these two related, but analytically different cases of co-constructions. Therefore, in the first example, we illustrate the “basic structure” of argumentative co-constructions primarily on an argumentative-structural level (grade 2); in the second example, co-constructions become additionally visible on the morpho-syntactical level (grade 4). With the third example, we show how the basic structure is extended by further argumentative elements (= “extended structure”, Kreuz, 2021) (grade 4).^4^

### Completion of argument structures: “Bush knife” – “to chop wood”

(DIL = Dilan; NAT = Natale, grade 2).
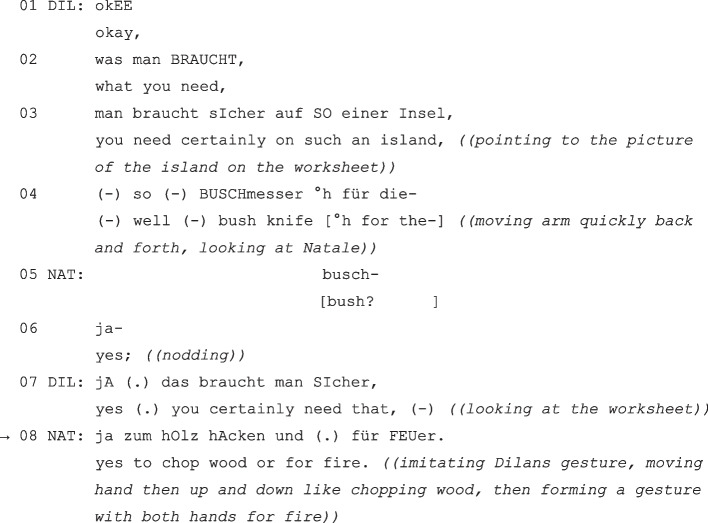


In this sequence, Dilan expresses his opinion about what is necessary to survive on a desert island (“what you need, you need certainly on such an Island, well bush knife for the”, lines 02–04). He expands his opinion suggestively by giving reasons: the reasons are introduced verbally (“for the”, line 04), but then continued with the help of illustrative gestures (Heller, 2012, p. 90), which represent the use of the bush knife with a gesture. Natale interrupts Dilan’s statement and repeats the proposition affirmatively (on repetitions to establish participation see Arendt & Zadunaisky Ehrlich in this volume) and ratifies it with an affirmative particle (line 06).

Even though the topic could be concluded by the clear consensus of the two children (cf. lines 06 and 07), the children remain on the topic of conversation and Natale participates co-constructively in Dilan’s suggestion. He adds a justification in line 08, which refers to the way the bush knife is used, which is only gesturally indicated (“yes to chop wood for fire”). Due to the close thematic reference to the previous utterance, Natale can elliptically supplement the utterance with a justification (while – and this will be different in example 2 – this immediately preceding utterance is syntactically complete). His justification both represents the second part of a co-constructed assertion-justification structure (1. “bush knives are needed” → 2. “to chop wood”) and it serves as a verbal explication of Dilan’s non-verbally produced justification (1. moving hand then up and down like chopping wood → 2. “to chop wood”). This sequence shows how Natale cooperatively participates in Dilan’s turn and verbalizes what the latter only vaguely hints at with his “argument” merely indicates through non-verbal illustrations. In the interpretation of this sequence, it must be considered that Dilan is a non-German native speaker.

In this example, there is never an obligation for a justification, but the first utterance is nevertheless followed up by a self-initiated justification with expansion and explication. It is conceivable that Natale sees himself in the role of a learning partner and offers ‘scaffolding’ for Dilan’s utterance. This reveals an acquisition-supportive pattern in shared peer interactions (see also Arendt, 2019a): It is possible that Natale recognizes an insufficiently explicated “head act” (Kyratzis et al., 2010) in the incomplete justification to build shared knowledge and secure mutual understanding. The argumentative co-construction reinforces consensus and *demonstrates*, rather than merely claims, understanding (Sacks, 1992), which is an important move in cooperative learning settings or joint solution finding.

The second example comes from a fourth-grade class where, in addition to the content-argumentative level of a co-construction, the microscopic level of a morpho-syntactical co-construction reveals the synchronous utterance production of two speakers.

### Morpho-syntactical completion of arguments: “Bush knife because uh…” – “to open bushes”

(MIL = Milan; DAR = Dario, grade 4).
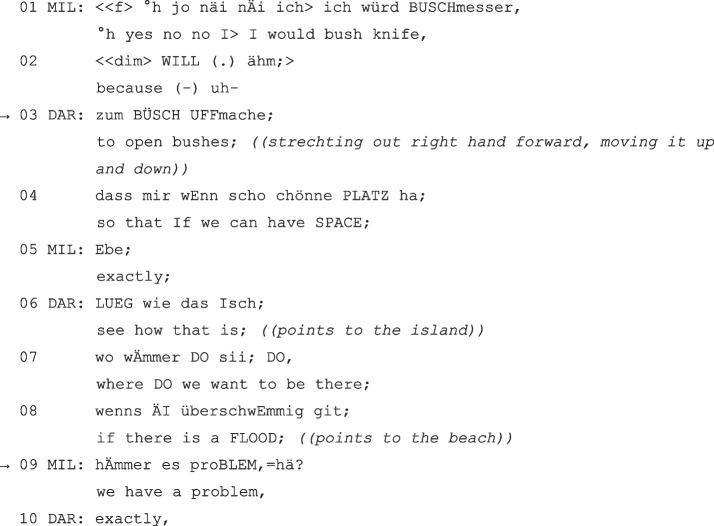


Milan proposes the bush knife in line 01 and marks a subsequent introduction of justifications by the conjunction “because” (line 02). However, he doesn’t complete his turn – marked by the prospective repairs (Liddicoat, 2007, p. 17; Pfeiffer, 2017; Schegloff et al., 1977, p. 363), the pause and the hesitation signal (e.g., Clark & Fox Tree, 2002), and the softening voice (“yes no no I I would bush knife because uh”, lines 01–02). In terms of the preference for action and representation progressivity (Deppermann, 2008; Mondada, 2011; Schegloff, 2007), a relevance to finish the incomplete turn emerges. This is taken over by Dario in line 03 by “nesting” (Mazeland, 2009, p. 202; cf. also Günthner, 2012, p. 9) a suitable justification in the previous utterance structure and thus completing the “syntactic gestalt” (Auer, 2005) (“to open bushes”, line 03). On the morpho-syntactical level of co-constructions, this is rather to be regarded as a special case since the syntactic connection is not formally completely appropriate. Nevertheless, this type of syntactic connection does not seem to be atypical for oral communication (cf. also epistemic ‘because’ for the German language, Feilke, 2015) and can be regarded in a broader sense as the second part of a “syntactic gestalt”. He thus contextualizes, on the one hand, his “close alignment with the conversational progress and the construction of the interlocutor, but on the other hand […] also [his] (partly concurrent) experience regarding the issue at hand” (Günthner, 2012, p. 6, our translation). His contribution is also coherent with Milan’s proposition on the content level, as he follows up with an aligned pro-justification (also illustrating it gesturally) and gives the proposition argumentative meaning (“see how that is, where do we want to be there, if there is a flood”, lines 06–08).

In line 09, another morpho-syntactical co-construction of Dario’s ‘if–then structure’, uttered by Milan, follows. Here, however, the completion takes place after a syntactically completed utterance, yet Milan treats the turn as needing completion and interprets the second part of Dario’s utterance (“if there is a flood”, line 08) as a hypotactic new starting point of a conditional structure to be completed. Thus, he completes this syntactic structure with the conclusion of the condition (“we have a problem”, line 09).

The sequence not only shows how the children continue each other’s argumentation although there is already consensus (Dario from line 06; cf. also “consensual” argumentation, Doury, 2012), but also how they both co-constructively complement or complete each other’s arguments in close syntactic connection (lines 02–03) and continue or expand them in a supporting manner (Dario from line 06, Milan line 09). The children thus not only show a simple agreement, but at the same time indicate an “affiliation with the perspective of [the] counterpart regarding the facts portrayed” (Günthner, 2012, p. 8, our translation; on “affiliation” see Arendt & Zadunaisky Ehrlich in this volume). The example further illustrates how the syntactic construction of the “first pair part” (Schegloff, 2007) is used to bring about a “syntactic gestalt” closure and at the same time to add a complete justification on the content level (lines 08-09).

Both morpho-syntactical co-constructions and elaborations (by the same or a third speaker) are more frequent in grade 4 and grade 6. This will be emphasized in the last example.

### Expansion of argument structures: “Lighter” – elaboration of argumentation

(TEV = Tevin; JUL = Julia; SIM = Simon, grade 4).

Finally, we’ll show an example in which not only two but three of the children develop their arguments through backings or supports (= extended structure). So, it is a very elaborated sequence that is co-constructed on several levels: In addition to the levels of basic argumentative-structural and morpho-syntactical co-constructions, co-constructions with further argumentative elements as well as repetitive and parallel sentence/word structures, gestures, eye contact and body orientation play an important role and can be considered as further indicators of securing mutual understanding and synchronicity.

The conversation is already concluded, and the children are in the final (consensual) understanding of their decision: The “matches” are repeatedly confirmed by all three children (lines 01–03). Nevertheless, the ‘head act’ is argumentatively expanded, and Simon co-constructs a justification.
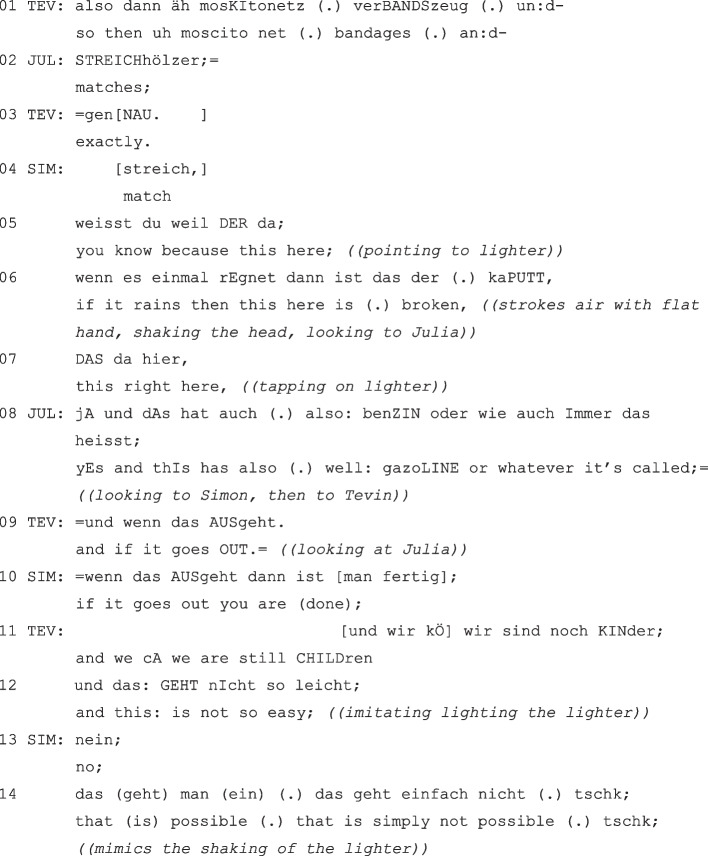


Tevin opens the sequence and lists the chosen objects again in co-construction with Julia (“so then uh mosquito net bandages and matches”, lines 01, 02). This develops into a longer co-constructed chain of common justifications and supports against the lighter. Simon is the first to co-constructively add a linguistically marked reason (“you know, because this there, if it rains, then this here is broken”, lines 05–06). He addresses this argument to Julia by means of eye contact (Hartung, 2001, p. 1351; Levinson, 1988, p. 179). Accordingly, Julia adds a second reason, which now refers to the properties of the lighter (“yes and this has also well gazoline or whatever it’s called”, line 08). Consensus is again indicated by both the agreeing particle “yes” and the second supporting justification.

Through the additive justifications of Simon and Julia, the consensus is not only “claimed”, but “demonstrated” several times (Sacks, 1992; cf. also Weatherall & Keevallik, 2016, p. 167) and thus intensified. Nevertheless, Tevin proceeds to another explicative conclusion (“and if it goes out”, line 09). However, he does not complete the unit of meaning, but only provides the first part of a conclusive set of conditions. Despite the interruption, this has argumentative and substantive force, which makes Julia’s reasoning plausible (in the sense of “the lighter can go out quickly”). The children do not leave it at that, however, but complete the elliptical antecedent through a morpho-syntactical completion (cf. also “conditional reasoning”, Lerner, 1991). For this, Simon takes up the first part of Tevin’s support again in form of an echo, but then continues it with the second part of the conditional (“if that goes out you’re (done)”, line 10).

The consensual chain of argumentation of the extended structure that has already emerged is now supplemented by Tevin with a third justification, which is also connected by “and” (“and we are still children”, line 11). Tevin ends his reasoning with the implied support “and this is not so easy” (line 12).

After this consensual chain of argumentation, however, the argumentation is still not complete; instead, Tevin’s justification is supported by Simon and even intensified in terms of both content and gesture (“that is simply not possible”, line 14).

The syntactic and lexical similarities of the children’s justification and support (cf. “format tying” according to Goodwin, 2006) shows how strongly speaker-differentiated utterances are marked as belonging to each other and the children also support their “joint fantasizing” (Kotthoff, 2007) through linguistic forms. This also becomes clear in the syntactic ‘adoptions’ or exact continuations of the previous utterances. Since the individual contributions are produced in rapid succession and syntactically condensed, the sequence shows a dynamic takeover of the rights to speak. On the argumentative level, the fact that argumentative supports are produced at all shows a high contextual sensitivity on the part of the children and their awareness of collaboration in cooperative learning settings. Interacting appropriately to the situation can be regarded as a central conversational competence (cf. school curricula, where aspects like these are mentioned).

The children make a great effort to co-constructively explicate their utterances at different levels and to intensify consensus through elaborate argumentation. “Consensual argumentation” becomes very clear here as well as multiple functions of co-constructed reasoning.

## Discussion

We summarize our research interests and analysis about synchronization performances as an interactional practice for taking part in solution-oriented group discussions among peers by first addressing our research questions and then discussing questions related to language teaching.

1. Which synchronization performances (especially on argumentative-structural level) can be reconstructed in co-constructed negotiation processes within argumentative peer conversations and how do these co-constructions serve in taking part in the current conversation sequence?
  Our data show that synchronization performances in the form of co-constructions can mostly be found in consensual sequences, where the children support each other in developing an argument. These co-constructions, as the three examples showed, range from basic structures (proposition + justification) to extended structures (proposition + justifications + backings). Especially in the case of extended structures, it becomes obvious that it is necessary not only to consider morpho-syntactical co-constructions, but also argumentative-structural ones. The co-constructions in these episodes include morpho-syntactical completions of syntactic “gestalts” (Auer, 1996) (co-constructions in a narrow sense), which are always contributions on the argumentative-structural level as well, but also elliptical verbal explications of gestures of a first speaker (example 1) or expansions on an argumentative-structural level (example 3), which do not necessarily close a syntactic gestalt, but elaborate and expand the argument the children are working on together. Still, these following turns are marked strongly as belonging to the preceding ones by format tying and syntactic adoptions. We argue therefore to consider them as co-constructions as well.
  Next to the observation that morpho-syntactical and argumentative-structural co-constructions are often closely connected, we could also show that verbal and gestural activities complement each other in co-constructed argumentative episodes and support the other’s arguments or even serve as (accepted) justifications.
  Our analysis of three examples showed that co-constructions are an index of a high-involvement style as they require a high degree of contextual sensitivity; they also indicate affiliation (Arendt & Zadunaisky Ehrlich in this volume), as they demonstrate consensus. Indicating involvement as well as demonstrating affiliation are both important features of taking part in a conversation and are important moves in cooperative learning settings.
2. Which pragmatic functions of co-constructions can be reconstructed in the data and how is the ‘problem’ thereby processed/resolved?
  In our research tasks, the children had the challenge (or ‘problem’) to find a consent. In our examples, we can see on an argumentative-structural level that co-constructions help to intensify and consolidate consensus by elaborating arguments together and making plausibility checks. Co-constructions allow exploring a topic together and building shared knowledge, securing mutual understanding, and reinforcing consensus by demonstrating (and not only claiming) it (cf. also Kreuz, 2021). The latter is the case when co-constructed utterances complete or expand argumentations from a first speaker. Then, we could also see that co-constructions are supporting “joint fantasizing” (Kotthoff, 2007). In the end, they contribute to consolidate a decision. Co-constructions are also relevant in a social dimension, as they mark group membership and demonstrate harmony as well as mutual understanding, which are goal-oriented behaviors to work on a topic in solution-oriented, cooperative learning settings.
  In one example, we also could see that one child explained in words what the other, who does not speak German as first language, has gestured. In this case, the second child is seeing itself as a learning partner and the co-construction is an acquisition-supportive pattern, also securing mutual understanding. This is also the case for morpho-syntactical co-constructions. They are often used to help another child with word search by completing incomplete propositions into a complete argument. Therefore, also morpho-syntactical co-constructions usually work on the argumentative-structural level too, as they help constructing a shared idea and thus contribute to a joint, cooperative knowledge production.
3. What differences can be observed between different ages?
  We selected examples from grade 2 and grade 4 because differences in the use of co-constructions are more evident between these two age groups than in comparison to grade 6 (for a detailed age comparison see Kreuz, 2021). Going beyond our examples discussed above, and regarding our entire corpus, in grades 4/6 we could observe among others:
  a greater variety of *contexts* and conditional relevance for co-constructions, e.g., children also use the context of consensus to co-construct arguments together.
  fine tuning in *linguistic linking*, e.g., we could identify more synchronized “nesting” (Mazeland, 2009) in the syntactical structures of the other participants in the sense of morpho-syntactical co-constructions trough completions of first pair parts.
  increasing *elaborations* of argumentative sequences, e.g., addition of examples and backings for one’s others arguments (extended structure)
  difference in *functions* of using co-constructions, e.g., social functions like demonstrating understanding and mutual harmony.

As the data analysis reveals co-constructions are argument-structural, linguistic, and multimodal ‘key sites’ of shared thinking through synchronization and adjustments (c.f. Goetz & Shatz, 1999) on different levels. This shows that the giving of justifications is not only reciprocally mirrored or adopted (as e.g., in action-opposition sequences) (Arendt, 2019a, p. 83), but maintains a consensual-argumentative conversational sequence in shared commonality and participation.

At the same time, the sequential micro-analysis of argument structures by the means of Conversation Analysis provides an insight into age-related practices in solution-oriented group discussions. In terms of educational contexts, these results – within an understanding of *interactive* oral argumentations (see Sect. “Research context: Oral argumentation and co-constructions”) – are of interest when it comes to assess argumentative activities of students. It can also be helpful for teachers to elicit requirements for children’s argumentation skills and to design tasks conducive to learn to argue and develop assessment tools accordingly. On the basis of our results, we argue that group discussions among peers can be legitimized as learning contexts for oral argumentation skills (cf. also Arendt, 2019a), because moments of implicit peer-learning of conversational practices were revealed, e.g., through model utterances as ‘online help’ (for subsequent utterances). Peer discussions, especially consensus-oriented ones, support collaborative social and cognitive learning (cf. also Baker et al., 2019), as in co-constructed argumentations the children offer each other room for learning, support each other and practice to relate their utterances context sensitive to each other, which is described as one of the ‘dialogic competences’ in school curricula. With the analysis of our data, a description tool can be developed that provides information about the practices of the students and can be compared with the competence descriptions of the curriculum for observing and assessing argumentative performances in school.

## Footnotes

1 Oral Argumentation Skills at School: Contexts, Requirements, Acquisition Processes (Argumentative Gesprächskompetenz in der Schule: Kontexte, Anforderungen, Erwerbsverläufe), funded by the Swiss National Foundation, grant number 149382.

2 We also coded the conversations with a focus on argumentative moves, always considering the local conversational context. This coding also allows for quantitative analysis, see Luginbühl et al. (2021).

3 This is in line with Domberg et al. (2017), who observe that children 5–7 years old produced not only more arguments, but also more two-sided arguments. Iordanou & Kuhn (2020) on the other hand observe in discussions of young adolescents that adversarial argumentation, employing the aim to persuade, is a more productive means, compared to collaborative argumentation.

4 The children's discussions were held in Swiss German and/or Standard German. The translation into English is as authentic as possible to the original.
